# Methodological and analytical considerations for intra-operative microdialysis

**DOI:** 10.1186/s12987-023-00497-2

**Published:** 2023-12-19

**Authors:** Cecile Riviere-Cazaux, Karishma Rajani, Masum Rahman, Juhee Oh, Desmond A. Brown, Jaclyn F. White, Benjamin T. Himes, Ignacio Jusue-Torres, Moses Rodriguez, Arthur E. Warrington, Sani H. Kizilbash, William F. Elmquist, Terry C. Burns

**Affiliations:** 1 Department of Neurological Surgery, Mayo Clinic, 200 First St. SW, Rochester, MN 55905 USA; 2 Brain Barriers Research Center, Department of Pharmaceutics, College of Pharmacy, University of Minnesota, Minneapolis, MN USA; 3 Neurosurgical Oncology Unit, Surgical Neurology Branch, National Institutes of Neurological Disorders and Stroke, National Institutes of Health, Bethesda, MD USA; 4 Department of Neurological Surgery, Wake Forest Baptist Health, Winston-Salem, NC USA; 5 Department of Neurological Surgery, Montefiore/Albert Einstein College of Medicine, Bronx, NY USA; 6 Department of Neurology, Mayo Clinic, Rochester, MN USA; 7 Department of Medical Oncology, Mayo Clinic, Rochester, MN USA

**Keywords:** Glioma, Microdialysis, Metabolomics, IDH, Pharmacokinetic, Mass spectrometry

## Abstract

**Background:**

Microdialysis is a technique that can be utilized to sample the interstitial fluid of the central nervous system (CNS), including in primary malignant brain tumors known as gliomas. Gliomas are mainly accessible at the time of surgery, but have rarely been analyzed via interstitial fluid collected via microdialysis. To that end, we obtained an investigational device exemption for high molecular weight catheters (HMW, 100 kDa) and a variable flow rate pump to perform microdialysis at flow rates amenable to an intra-operative setting. We herein report on the lessons and insights obtained during our intra-operative HMW microdialysis trial, both in regard to methodological and analytical considerations.

**Methods:**

Intra-operative HMW microdialysis was performed during 15 clinically indicated glioma resections in fourteen patients, across three radiographically diverse regions in each patient. Microdialysates were analyzed via targeted and untargeted metabolomics via ultra-performance liquid chromatography tandem mass spectrometry.

**Results:**

Use of albumin and lactate-containing perfusates impacted subsets of metabolites evaluated via global metabolomics. Additionally, focal delivery of lactate via a lactate-containing perfusate, induced local metabolic changes, suggesting the potential for intra-operative pharmacodynamic studies via reverse microdialysis of candidate drugs. Multiple peri-operatively administered drugs, including levetiracetam, cefazolin, caffeine, mannitol and acetaminophen, could be detected from one microdialysate aliquot representing 10 min worth of intra-operative sampling. Moreover, clinical, radiographic, and methodological considerations for performing intra-operative microdialysis are discussed.

**Conclusions:**

Intra-operative HMW microdialysis can feasibly be utilized to sample the live human CNS microenvironment, including both metabolites and drugs, within one surgery. Certain variables, such as perfusate type, must be considered during and after analysis.

*Trial registration* NCT04047264

**Supplementary Information:**

The online version contains supplementary material available at 10.1186/s12987-023-00497-2.

## Background

Few methods exist via which the live central nervous system (CNS) can be sampled and analyzed in situ, including in the presence of a brain tumor. Gliomas are primary malignant brain tumors that inevitably recur and for which no cure currently exists [1]. The glioma and its extracellular microenvironment remain poorly understood as the tumor is only immediately accessible in the operating room, at the time of resection [2]. Studies in gliomas, as well as other CNS diseases, such as Alzheimer’s Disease and Parkinson’s Disease, often rely on animal models that incompletely recapitulate the pathology and therapeutic resistance of the disease [3–6]. Understanding and sampling this biology in situ could enable therapeutic discoveries guided by the patient’s own live disease.

Microdialysis is a technique that enables sampling of candidate biomarkers from the tissue microenvironment [7]. Perfusate flows through the sampling catheter that has a semipermeable membrane with a set molecular weight cut-off. The concentration gradient between the parenchyma and the perfusate enables extracellular parenchymal analytes to diffuse across the membrane and into the recovered microdialysate. Microdialysis has been deployed in traumatic brain injury studies to identify biomarkers of tissue health and damage, including glucose [8, 9], *n*-acetylaspartate (NAA) [10], lactate/pyruvate ratio [9, 11], and glutamate [12]. In glioma and other CNS diseases, microdialysis has been utilized for pharmacokinetic studies to evaluate local CNS free drug levels [13–15]. More recently, we and others have utilized microdialysis in gliomas to evaluate the extracellular metabolic and cytokine/chemokine microenvironment of these tumors [16–22]. We recently reported findings from our intra-operative high molecular weight (HMW) microdialysis trial (NCT04047264) [23]. This work identified a highly conserved signature of blood–brain barrier disruption across patients with enhancing tumors, enriched for plasma-derived analytes. As a first-in-human intra-operative study of HMW microdialysis, multiple lessons were learned from each patient about the potential pitfalls and opportunities of intra-operative HMW microdialysis.

Herein, we report on (or “confess”) these lessons and surreptitious insights obtained during intra-operative HMW microdialysis. We discuss clinical, radiographic, and methodologic considerations to optimize feedback from the tumor in a relatively short period of time during ongoing resection. The impact of different perfusate components, including lactate and albumin, was retrospectively evaluated. While not intentional, we demonstrate the metabolism of focally delivered lactate via reverse microdialysis from Lactated Ringer’s-containing perfusate into pyruvate. This finding suggested the potential to focally alter the local metabolic microenvironment via reverse microdialysis for future pharmacodynamic studies. Finally, although we did not expect that untargeted global metabolomic analyses would detect multiple drugs, we demonstrate that 10 min (20 µL) worth of sampling time can reflect each patient’s pharmacologic intra-operative management inclusive of 5 distinct drugs. In sum, our experiences with intra-operative HMW microdialysate highlight variables for methodological and analytical consideration, as well as the substantial utility of this method for sampling the live human CNS microenvironment, in situ.

## Methods

### Patient cohort and study design

Patients provided written informed consent to participate in NCT04047264, “Feasibility of Intraoperative Microdialysis During Neurosurgery for Central Nervous System Malignancies.” Study procedures, including use of M dialysis HMW catheters and variable flow rate pump (M dialysis 107) under an investigational device exemption, were approved by the Mayo Clinic Institutional Review Board. Inclusion criteria included adults that were 18 years or older undergoing a clinically indicated resection for a known or suspected glioma. No patients or catheters were excluded for analyses. When feasible, paired intracranial cerebrospinal fluid (CSF) was obtained for each patient and stored under Mayo Clinic’s Neuro-Oncology biorepository.

### Clinical, radiographic, and intra-operative considerations for microdialysis

**Fig. 1:**
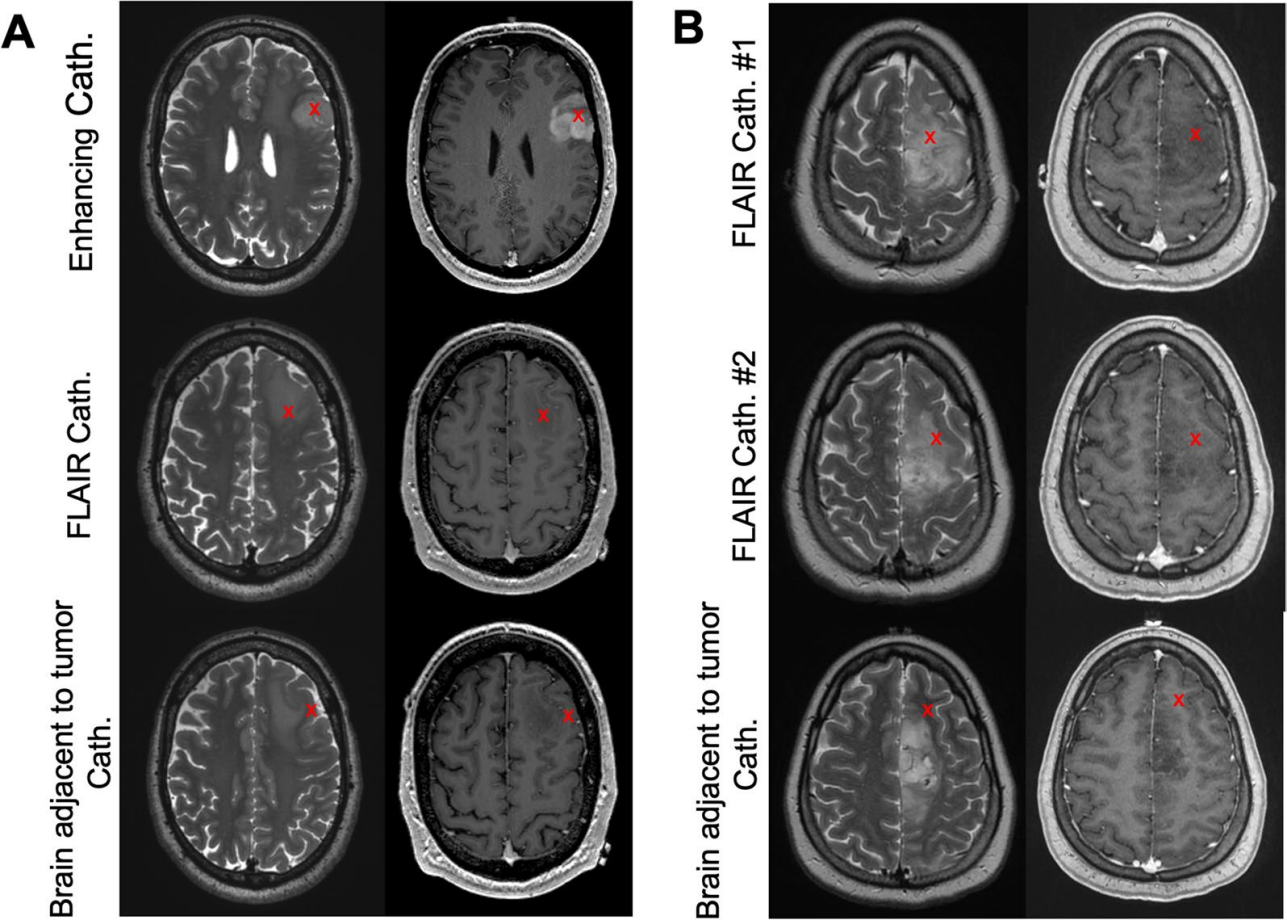
Pre-operative magnetic resonance imaging (MRI) was utilized to define microdialysis catheter placement in radiographically diverse regions. **A** In patients with contrast-enhancing tumors, microdialysis catheters were placed in enhancing and non-enhancing tumor, as well as brain adjacent to tumor. **B** In patients with non-contrast enhancing tumors, two microdialysis catheters were placed in non-enhancing tumor and one in brain adjacent to tumor. Left: T2 FLAIR; right: T1-weighted post-contrast sequence; X indicates location of catheter placement

Patients were recruited to participate on this trial (NCT04047264) who were undergoing a clinically indicated resection for a known or suspected glioma. Fifteen cases, including one patient’s primary and repeat resection, were included in this study, spanning a total of 44 analyzed catheters (Table 1). Tumors selected for microdialysis were often large enough to enable sampling of multiple areas in the tumor while resection was ongoing within other portions of the tumor. An additional consideration included whether relatively normal brain adjacent to tumor would be exposed via the craniotomy without altering the planned surgical approach. Pre-operatively acquired magnetic resonance imaging (MRI) with gadolinium contrast was utilized to decide which areas would be sampled by the microdialysis catheters. In patients with enhancing high-grade gliomas, at minimum one each of enhancing tumor (bright on T1w-post-gadolinium), non-enhancing tumor (FLAIR positive), and relatively normal brain adjacent to tumor were sampled (Fig. 1A). In patients with completely non-enhancing, likely lower-grade gliomas, at minimum two different non-enhancing (FLAIR positive) and one relatively normal brain adjacent to tumor areas were sampled (Fig. 1B). When planning the catheter trajectory and depths, care was taken to ensure that no sulci were crossed, and that the full length of the catheters’ membranes would be within tissue (10 mm minimum). The depth of catheter placement was then determined based on the path to the target of interest. Catheter trajectories were arranged to prevent any collisions if they were near each other. Trajectories were planned for minimal interference with the surgical approach. These trajectories sometimes had to be adjusted immediately prior to insertion based on tumor density or surgical exposure, using the neuronavigation system. For later cases, a straight Rhoton microdissector was utilized to create a path through fibrous tumor tissue based on registration to the neuronavigation system.

**Table 1:** Clinical information on the patient cohort

Patient ID	Diagnosis	Number of catheters and lengths (X–Z)	Perfusate utilized	Awake or asleep?
Oligo^2^	Oligodendroglioma, grade 2	3 (X and Z: 10 mm; Y: 20 mm)	LR with 3% Dextran 40	Awake
Oligo^3^1	Oligodendroglioma, grade 3	3 (X and Y: 20 mm; Z: 10 mm)	LR with 3% Dextran 40	Awake
Oligo^3^2	Oligodendroglioma, grade 3	3 (X–Z: 10 mm)	aCSF with 3% Dextran 500	Asleep
Oligo^3^3	Oligodendroglioma, grade 3	3 (X–Z: 10 mm)	3% Albumin A in Plasmalyte-A	Asleep
GemAstro^3^-mut	Gemistocytic astrocytoma, IDH-mutant, grade 3	3 (X–Z: 10 mm)	3% Albumin A in Plasmalyte-A	Asleep
Astro^4−mut^1	Astrocytoma, IDH-mutant, grade 4	3 (X–Z: 10 mm)	LR with 3% Dextran 40	Asleep
Astro^4−mut^2	Astrocytoma, IDH-mutant, grade 4	3 (X–Z: 10 mm)	3% Albumin A in Plasmalyte-A	Awake
Astro^4−mut^3	Astrocytoma, IDH-mutant, grade 4	3 (X–Z: 10 mm)	aCSF with 3% Dextran 500	Asleep
H3K27M-Astro^4−WT^	H3K27M-mutated astrocytoma, IDH-wild type, grade 4	3 (X–Z: 10 mm)	aCSF with 3% Dextran 500	Asleep
GBM^WT^1	Glioblastoma	3 (X–Z: 20 mm)	LR with 3% Dextran 40	Awake
GBM^WT^2/2B	Glioblastoma (2B: repeat surgery)	3/2 (X–Z: 10 mm/ X, Z: 10 mm)	aCSF with 3% Dextran 500	Awake
GBM^WT^3	Glioblastoma	3 (X–Z: 10 mm)	aCSF with 3% Dextran 500	Asleep
GBM^WT^4	Glioblastoma	3 (X–Z: 10 mm)	aCSF with 3% Dextran 500	Asleep
MolecGBM^WT^	Molecular glioblastoma	3 (X–Z: 10 mm)	aCSF with 3% Dextran 500	Asleep

These characteristics can also be found in our previously published manuscript from this same patient cohort [23]

*aCSF* artificial CSF, *LR* Lactated Ringer’s

We have previously reported our abbreviated methods for intra-operative microdialysis [23]. Three high-molecular weight microdialysis catheters (100 kDa, 71 High Cut-off Brain Microdialysis Catheter, M Dialysis) were utilized for 14/15 surgeries; two catheters were utilized in one surgery. Membrane length was typically 10 mm (ref: 8010320), but 6/44 catheters had a length of 20 mm (ref: 8010331). Different membrane lengths were utilized to determine whether (1) a longer membrane length increased metabolite recovery, as multiple prior studies have demonstrated proportionality between membrane surface area and recovery (reviewed here [24]), and (2) if longer length could be utilized to target larger homogeneous areas of tumor and brain. A variable flow rate pump (107 Microdialysis pump, M Dialysis) was utilized for each catheter and set to sample at a rate of 2.0 µL/min after an initial flush period to obtain sufficient volumes for analysis. Absolute recovery is proportional to perfusate flow rate up to 2 µL/min [24].

Each syringe was filled with perfusate, which was either: (1) 3% 500 kDa Dextran with artificial CSF (Dextran MW 500 kDa 3%; Reference: 8050151, Perfusion Fluid CNS Dextran for Microdialysis, M Dialysis; 8/15 cases), (2) 30% 40 kDa Dextran (Pfizer, 10% LMD in 0.9% NaCl) with Lactated Ringer’s solution (4/15 cases), or (3) 3% albumin (Grifols Albutein ® 25% 20 mL) in Plasmalyte-A Injection pH 7.4 (3/15 cases). Perfusates typically contain a high molecular weight oncotic solution, such as albumin [24–26] or dextran (more commonly) [27, 28], that is too large to pass through the membrane and can thus increase analyte recovery by establishing an oncotic gradient between the perfusate and tissue. The base of perfusion fluid is usually a solution that is isotonic with the tissue containing sodium, chloride, and other ions, to minimize the amount of transfer between the perfusate and the tissue, hence why artificial CSF and Plasmalyte-A (a sterile nonpyrogenic isotonic electrolyte solution infused in the OR) were utilized [24]. Ringer’s has also previously been utilized in microdialysis as it contains sodium, chloride, bicarbonate and other ions [28]. While we had intended on using a Ringer’s solution when Plasmalyte-A or artificial CSF were not available, we instead utilized Lactated Ringer’s in four cases, as we did not yet recognize the potential impacts that the addition of lactate could have on microdialysate.

**Fig. 2:**
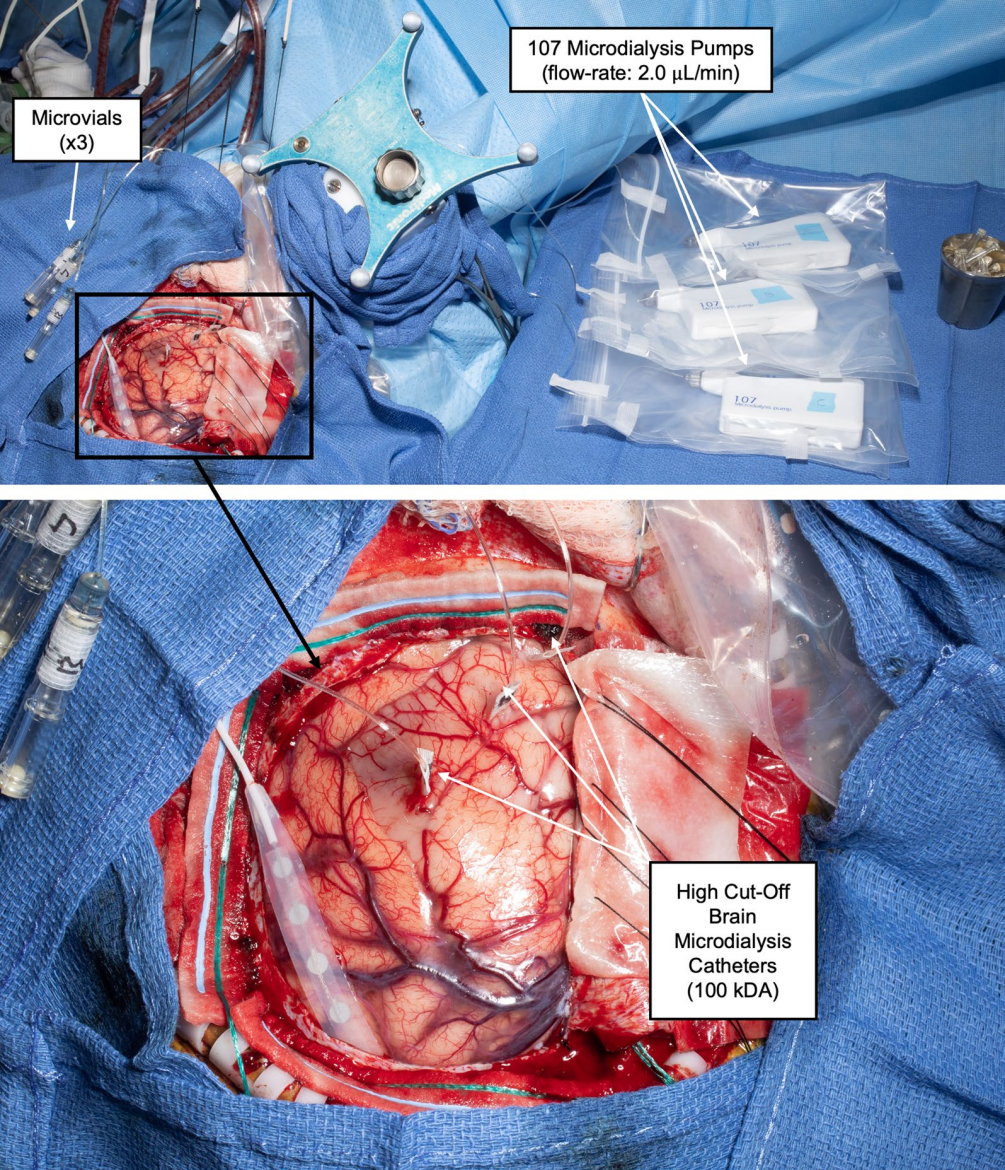
Intra-operative microdialysis set-up. 107 Microdialysis variable-rate pumps are connected to high cut-off brain microdialysis catheters (100 kDa) and microvials to sample three radiographically diverse regions during clinically indicated brain tumor resections, including enhancing or non-enhancing tumor and/or brain adjacent to tumor, as described in Fig. 1 (top: overview; bottom: close-up of resection field)

Intra-operatively, two or three variable flow rate 107 microdialysis pumps were placed on a Mayo stand close to the operating field (Fig. 2A). Catheters were flushed with the perfusate for at least twenty minutes and were then inserted via stereotactic navigation using the radiographically identified sampling locations, sampling at a flow rate of 2.0 µL/min with aliquots collected every 20 min. Tubing was carefully secured adjacent to the exposure to mitigate catheter migration and minimize interference with the surgical resection. Concurrent tumor resection during microdialysis minimized prolongation of surgery by the microdialysis procedures. A steristrip was placed on each catheter at the desired cortical depth; close attention was needed to ensure catheter did not dislodge from their desired depth during tumor resection. 40% (n = 6/15) of the cases were performed with awake language and neurocognitive mapping conveniently allowing sampling to begin while initial cortical mapping was ongoing. Of note, although 40 µL of microdialysate should have been collected from each 20-min fraction, splitting of the aliquots intra-operatively beginning with case GBM^WT^2B demonstrated a recovery of 30–35 µL for most catheters, suggesting some net fluid efflux across the semipermeable membrane. In all cases, 3–4 aliquots were obtained from each patient’s catheter; in one case for a non-enhancing insular glioma (MolecGBM^WT^), 7 aliquots, representing over 2 h of sampling time, were successfully collected from each catheter. Once the sampling area needed to be surgically resected, catheters were removed, and a biopsy obtained at the site. Microdialysate aliquots were stored at − 80 °C until analysis. No complications occurred that were attributable to microdialysis.

### Targeted analysis of D and L-2-hydroxyglutarate and lactate

Quantitation of both D and L-2-hydroxyglutarate in the four timed aliquots from each of patient Astro^4−mut^1’s catheter, as well as lactate in the ten CSF samples, was performed by the Mayo Clinic Metabolomic Core Facility via liquid chromatography mass spectrometry, as previously reported [23].

### Untargeted metabolomic analyses and normalization

Samples were sent to Metabolon, Inc. for untargeted metabolomic analysis of 51 microdialysate samples in 3 batches. Samples included one aliquot from each of the 44 catheters sampling tumor or brain during the fifteen surgeries. Additional samples included five Dextran 500 flushes (1 in batch 1, 2 in batch 2, 2 in batch 3), one albumin flush, and one Dextran 40 flush. Two fractions each of the 15–20 µL aliquots were evaluated with reverse phase tandem ultra-performance liquid chromatography mass spectrometry. Fractions were evaluated via both positive and negative ion mode ESI, as well as hydrophobic interaction chromatography (HILIC) UPLC-MS/MS under negative ion mode ESI. Further details can be found in the supplementary methods of our prior work [23]. After extraction of raw data from UPLC-MS/MS, metabolites were identified from Metabolon’s extensive library based on retention index, mass-to-charge ratio (m/z), and chromatographic data. For samples where less than 20 µL of microdialysate were found, peak areas were scaled to 20 µL prior to other analyses. Data were batch-normalized, such that the median of each metabolite in each batch was 1.

### Relative loss of lactate

To calculate the “relative loss” of lactate from the perfusate, the following equation was used: $\frac{\mathrm{Cin}-\mathrm{Cout}}{\mathrm{Cin}}$, where C_in_ was the untargeted raw peak area for the Dextran 40/Lactated Ringer’s flush and C_out_ was the recovered raw peak area of lactate in each of the catheters [24]. The flush submitted for analysis was the initial flush fluid from patient Oligo^3^1. A linear correlation was performed on the targeted absolute quantification versus untargeted relative quantification of lactate from ten CSF samples prior to this calculation to confirm the performance of untargeted metabolomics for relative lactate quantification.

### In vitro microdialysis of CSF

To determine the impact of microdialysis on analyte recovery, a 100 kDa catheter (100 kDa, 71 High Cut-off Brain Microdialysis Catheter, M Dialysis; 10 mm; reference: 801820) was utilized to microdialyze room temperature CSF from two different patients, with a 107 Microdialysis variable flow rate pump. In both cases, intracranial CSF had been obtained from two patients with astrocytomas via ventriculoperitoneal shunts. CSF was spun down at 400G for 10 min at 4 °C prior to being aliquoted and stored at − 80 °C in cryovials. On the day of the experiment, CSF was thawed until it was at room temperature. After the flush cycle, the catheter was inserted into the CSF for microdialysis at a flow rate of 2 µL/min in 20-min fractions. The second fraction after the flush was frozen at − 80 °C until analysis. The CSF was then re-frozen at − 80 °C. Untargeted metabolomics was performed on both the microdialysate of CSF, as well as the CSF both prior to and after microdialysis.

### Heat maps

The heat maps in Fig. 4B and Additional file 1: Fig, S3 were generated based on the top 25 metabolites present in the albumin and Plasmalyte A flush using normalized data. The normalized peak areas were then evaluated for each of these 25 metabolites.

### Statistical and correlation analyses

Data are presented either as raw peak areas or normalized peak areas (as described above). Full raw and normalised peak area data tables for these studies were previously published [23]. MetaboAnalyst 5.0 was used for Pearson correlation in Fig. 4A using the 162 metabolites present in at least 90% of the 44 catheters. GraphPad PRISM 9.1 was utilized to generate all heat maps.

## Results

### Time course of fractions and normalization

The isocitrate dehydrogenase (IDH) mutation has profound effects on glioma biology and results in elevated production of the oncometabolite, D-2-hydroxyglutarate (D-2-HG) [29]. One of the first questions we asked was whether there was a difference in the global extracellular metabolome of IDH-mutant versus IDH-wild type patients. Multiple aliquots were obtained for each patient. Given the elevated flow rates utilized for this short sampling period, we wished to determine whether microdialysate fully equilibrated with the interstitial fluid within the surgical sampling period based on relative change between successive aliquots. The first patient enrolled in the microdialysis trial had a grade 4 IDH-mutant astrocytoma and three aliquots acquired after the first “flush” aliquot (#1). As such, 2-HG was assayed in the four aliquots from three catheters via liquid chromatography-mass spectrometry to evaluate the recovery of this metabolite over time (Additional file 1: Fig. S1). In the enhancing and non-enhancing tumor catheter, D and L-2-HG recovery peaked within the first twenty-minute fraction after catheter insertion (aliquot #2) (Additional file 1: Fig. S1A, B). D-2-HG was highest in the next (second) aliquot. The 3rd and 4th aliquots in the enhancing tumor contained 24.6 and 23.5% of maximum. In non-enhancing tumor, the 3rd and 4th aliquots contained 37.1 and 47.2% of maximum, respectively. The ongoing decrease in non-enhancing tumor tissue suggested that full equilibrium may not be obtained ever after 1 h of microdialysis—especially for highly abundant metabolites. Moreover, the high flow rate of 2 µL/min likely leads to substantially lower concentrations of analytes in recovered aliquots than originally present in the CNS interstitial fluid. Ultimately, due to sample size limitations and the number of aliquots collected per patient, aliquot #3, representing the 20–40-min interval following catheter placement, was utilized for global metabolomic analyses in all subsequent patient samples. Other samples were stored for future multi-omic and confirmatory targeted metabolomic analyses.

In preparation for untargeted analysis, we asked how metabolite recovery by microdialysis was impacted by the presence of the microdialysis membrane, when sampling at 2 µL/min across diverse analytes. To do so, we performed microdialysis of cerebrospinal fluid (CSF) and performed global metabolomic analyses on both the microdialysate and cerebrospinal fluid after microdialysis (Additional file 1: Fig. S2). Some metabolites were only present in CSF, and not detectable after microdialysis. Of metabolites detectable both in CSF and microdialysate thereof, the median metabolite abundance was 7.67 × and 7.71 × more times abundant in CSF as compared to microdialysate in the two patient samples for which this experiment was performed. A small subset of metabolites was higher microdialysate than CSF, or only present in microdialysate, perhaps suggesting that some analytes originated from the microdialysis membrane or tubing, named in the untargeted analyses, such as benzoate (4.72×), 4-chlorobenzoic acid (4.65×), and heptanoate (2.33×). Of note, lower abundancy in microdialysate could perhaps be due in part to some metabolites sticking to the microdialysis’ polysulfone membrane. The stability of metabolites in CSF after freeze-thawing or being at room temperature in the microvials may also impact measured differences in CSF versus microdialysis. Nevertheless, in combination with the 2-HG results above, these results collectively suggested that adequate recovery of metabolites was plausible using 2 µL/min HMW microdialysis.

**Fig. 3:**
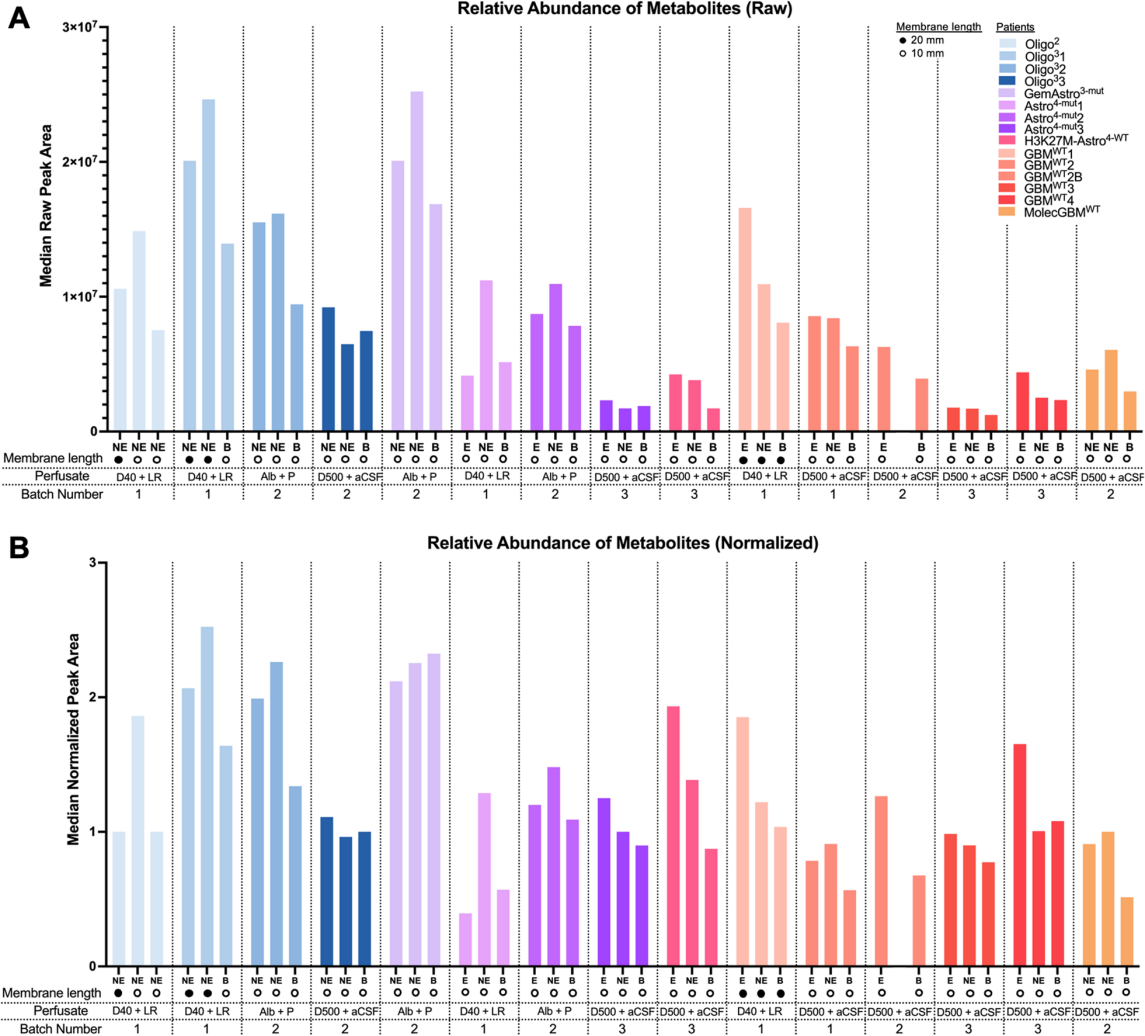
Batch, perfusate type, and membrane length do not impact the relative abundance of recovered metabolites recovered in microdialysate. The median **A** raw and **B** normalized peak area was calculated for each catheter based on the 162 metabolites present across at least 90% of catheters. Normalization was performed based on normalizing each metabolite to have a median of one in each batch. Batch number, membrane length (10 or 20 mm), or perfusate type are notated for each sample. (Perfusate: D40 + LR = Dextran 40 kDa and Lactated Ringer’s; Alb + P = albumin + perfusate; D500 + aCSF = Dextran 500 kDa + artificial CSF)

Global metabolomic analyses was performed utilizing the Metabolon platform via Ultra-performance liquid chromatography-mass spectrometry (UPLC-MS). Samples were analyzed across three different batches. Three different perfusate types (Dextran 500 kDa and artificial CSF, Dextran 40 kDa and Lactated Ringer’s, and albumin and Plasmalyte-A), with some batches containing samples obtained using multiple perfusate types. While most of the microdialysate catheters had 10 mm length membranes, six of the forty-four catheters had 20 mm length membranes. Both raw and normalized data were provided by Metabolon, Inc. Normalization was performed such that the median for each metabolite across any batch was one. To determine whether there was an impact of batch, perfusate type, and membrane length on the abundance of recovered metabolites, the median raw peak area was calculated for each catheter. No obvious impacts of these three factors on the median raw peak area could be discerned (Fig. 3A). The median raw peak area varied substantially across patients (range: 1.23 × 10^6^ to 2.52 × 10^7^). Normalization of the data decreased the variations in peak area across patients. As such, to permit comparisons across batches, most analyses utilized normalized data (Fig. 3B).

### Impact of perfusate composition on global metabolomics

**Fig. 4:**
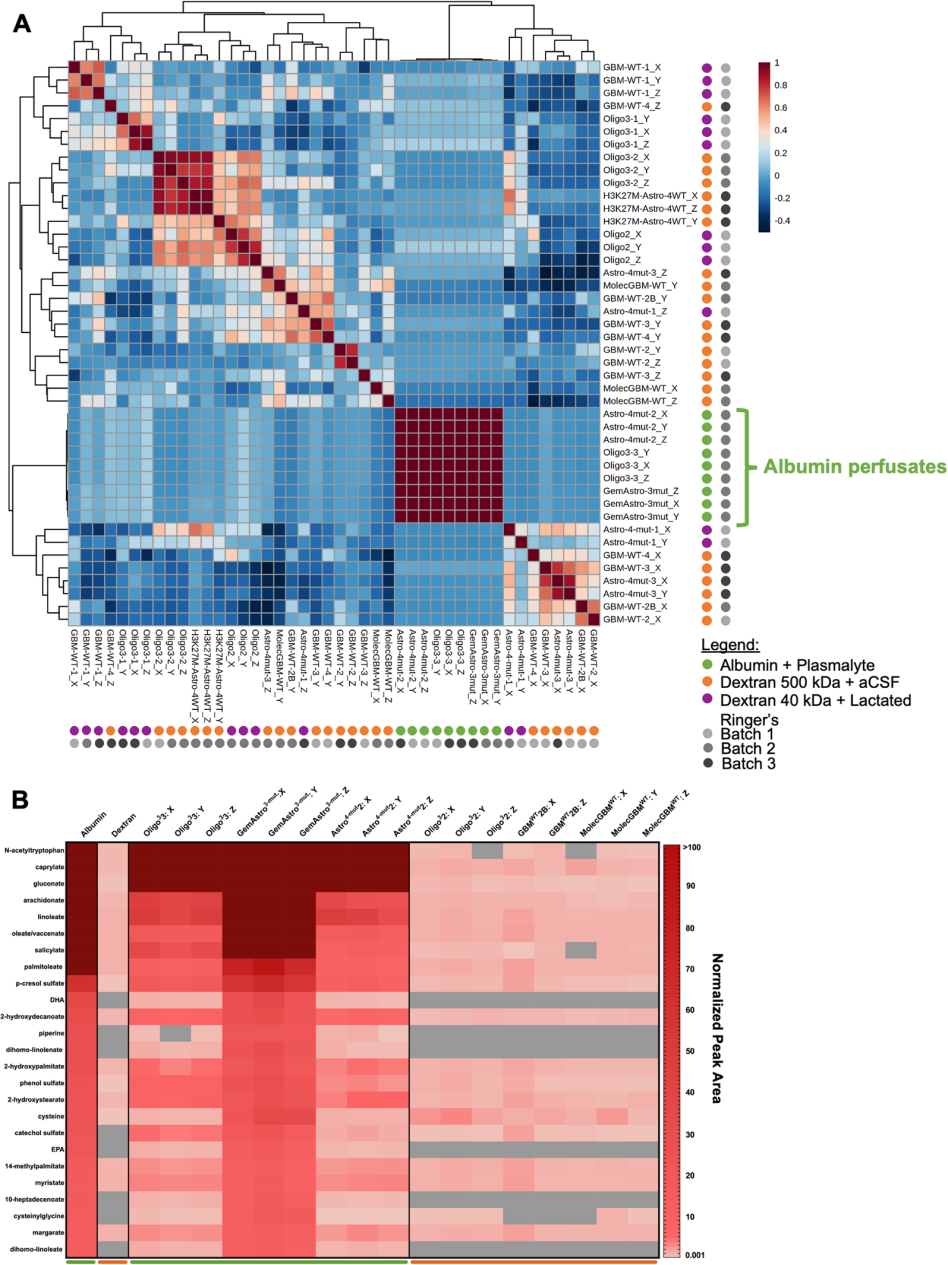
Albumin perfusate impacts microdialysate catheter clustering and a subset of metabolites. **A** Pearson correlation of the 162 normalized metabolites present in at least 90% catheters revealed clustering of microdialysates in which albumin was utilized as a perfusate. Batch and perfusate types are annotated. **B** A heat map was generated for the top 25 differentially abundant metabolites between the albumin versus dextran-containing microdialysates (both in batch 2) and these metabolites’ normalized peak areas in batch 2 patients; only the second batch is shown (see Additional file 1: Fig. S3 for full data across the 3 batches)

Perfusate utilized in microdialysis typically contains a high molecular weight compound, such as dextran, that can be utilized to increase the recovery of metabolites into the microdialysate by creating an osmotic gradient. Three different perfusates were utilized in these first fifteen cases: 500 kDa Dextran with artificial CSF (standard perfusate sold by M Dialysis), 40 kDa Dextran with Lactated Ringer’s, and albumin with Plasmalyte A. The latter two perfusates were utilized without advance knowledge of the potential impact that each perfusate could have on the global metabolome. Median raw peak area was not significantly different across albumin versus dextran-containing perfusate samples (Fig. 3B). Moreover, Spearman correlation analysis of the normalized data did not demonstrate sample clustering by perfusate [23]. Interestingly, however, when the normalized data were evaluated using Pearson correlation analysis, which assumes a linear correlation as compared to a monotonical correlation with Spearman, samples clustered cleanly based on the use of albumin-versus dextran-containing perfusate (Fig. 4A). The second batch of samples submitted for analysis contained both albumin and dextran-containing samples enabling direct comparisons. We ranked metabolites by relative abundance in an albumin-containing flush versus 500 kDA Dextran-containing flush samples (n = 2) from the same batch. Obvious differences between these perfusates are illustrated in the top 25 differentially abundant metabolites present in the albumin-containing flush (Fig. 4B; complete data including all batches in Additional file 1: Fig. S3). Albumin-containing samples included complex, large metabolites (namely fatty acids) such as n-acetyltryptophan, caprylate, gluconate, and arachidonate, some of which are contaminants that are pre-bound to commercial preparations of human albumin to stabilize it [30]. As such, a small subset of metabolites may be impacted by albumin-containing perfusate.

### Reverse microdialysis of lactate: inadvertent modulation of glioma metabolism, in situ

**Fig. 5:**
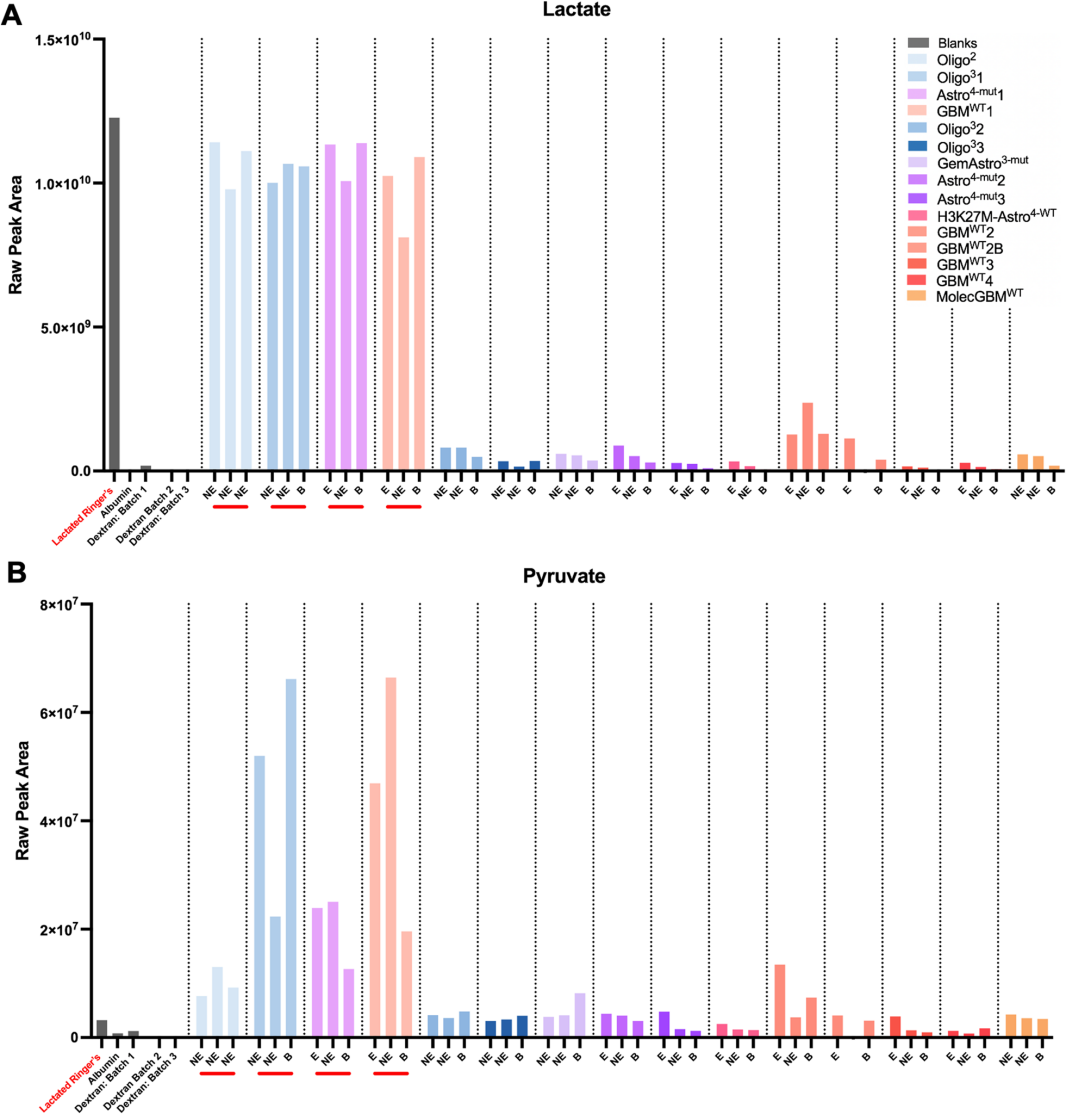
Focally delivered lactate via Lactated Ringer’s perfusate is metabolized to its derivative, pyruvate. The raw peak areas for **A** lactate and **B** pyruvate are shown. Lactate was focally delivered in patients for whom a Lactated Ringer’s-containing perfusate was utilized, indicated in red

Reverse microdialysis (rMD) has previously been performed to achieve focal delivery of specific compounds [21]. Perfusate typically consists of an isotonic solution. In four cases, Lactated Ringer’s was utilized as the only sterile isotonic solution available in the operating room. Although performed inadvertently, use of Lactated Ringer’s in the initial four cases enabled lactate to fall down its concentration gradient into the extracellular microenvironment, equating to net focal delivery of lactate via reverse microdialysis (Fig. 5A**)**. Raw peak areas confirmed relatively elevated lactate abundance in the recovered microdialysates for the four patients for whom dextran 40 + Lactated Ringer’s was utilized as a perfusate (Fig. 5A**;** average RFU: 1.05 × 10^10^ versus non-LR perfusates: 4.96 × 10^8^; 21.1 × difference lactate/non-lactate perfusate). Normalization was based on a median of 1 for each batch minimized the differences between the LR-based batch 1 and the other non-LR perfusate batches (Additional file 1: Fig. S4; average median-normalized RFU: 1.02 versus non-LR perfusates: 1.19; 0.85 × difference lactate/non-lactate perfusate). Focal delivery of lactate also provided an opportunity to estimate the relative recovery of HMW for lactate based on its loss. Using the dextran + Lactated Ringer’s flush sample, the relative loss of lactate from the perfusate into local tissue was 14.65% with a standard deviation of 7.61% (Table 2). Of note, these calculations were based on the relative quantification of lactate, which showed a strong correlation (R^2^ = 0.85) between untargeted and targeted metabolomic platforms (Additional file 1: Fig. S5). However, this relative loss of lactate is not a precise measurement; recovery may also vary based on the analyte’s unique properties.

**Table 2:** Relative loss of lactate from delivery via Lactated Ringer’s perfusate

Patient	Catheter	Untargeted (RFU)	Relative Loss (RL) of lactate: $\frac{{\mathrm{C}}_{\mathrm{in}}-{\mathrm{C}}_{\mathrm{out}}}{{\mathrm{C}}_{\mathrm{in}}}$
N/A	Blank: LR	1.23 × 10^10^	
Oligo^2^	X	1.14 × 10^10^	6.92%
	Y	9.79 × 10^9^	20.21%
	Z	1.11 × 10^10^	9.42%
Oligo^3^1	X	1.00 × 10^10^	18.42%
	Y	1.07 × 10^10^	13.04%
	Z	1.06 × 10^10^	13.76%
Astro^4−mut^1	X	1.13 × 10^10^	7.54%
	Y	1.01 × 10^10^	17.91%
	Z	1.14 × 10^10^	7.18%
GBM^WT^1	X	1.03 × 10^10^	16.43%
	Y	8.11 × 10^9^	33.85%
	Z	1.09 × 10^10^	11.14%
			Mean: 14.65% ($\pm$ 7.61% SD)

Lactate can be converted to pyruvate by lactate dehydrogenase and NAD + in the intracellular compartment (Fig. 5B). We noticed that pyruvate abundance was markedly higher in recovered microdialysate aliquots (average RFU: 3.04 × 10^7^) than LR-containing flush (3.20 × 10^6^; 9.51 × difference), or microdialysate samples obtained perfusate without LR (average RFU: 3.64 × 10^6^; 8.36 × difference). These data suggested that delivered lactate was rapidly incorporated into local metabolic pathways with increased pyruvate serving as a pharmacodynamic readout of lactate dehydrogenase activity, which is abundant in high-grade gliomas. In contrast, when evaluating citrate, the immediate derivative of pyruvate in the Krebs cycle, no differences in abundance could be observed across microdialysates with or without LR (Additional file 1: Fig. S6), suggesting that lactate had not been delivered for a long enough amount of time to impact further downstream pathways.

### Detection of multiple peri-operative drugs in 20 µL of microdialysate 

Multiple drugs and their metabolic derivatives were present within the metabolites identified in untargeted analysis. Patients routinely receive levetiracetam (Keppra), cefazolin (Ancef), and mannitol peri- and intra-operatively, while caffeine and acetaminophen are sometimes administered during awake tumor resections. Each was detected in appropriate patient’s samples from 20 μL of microdialysate.

**Fig. 6:**
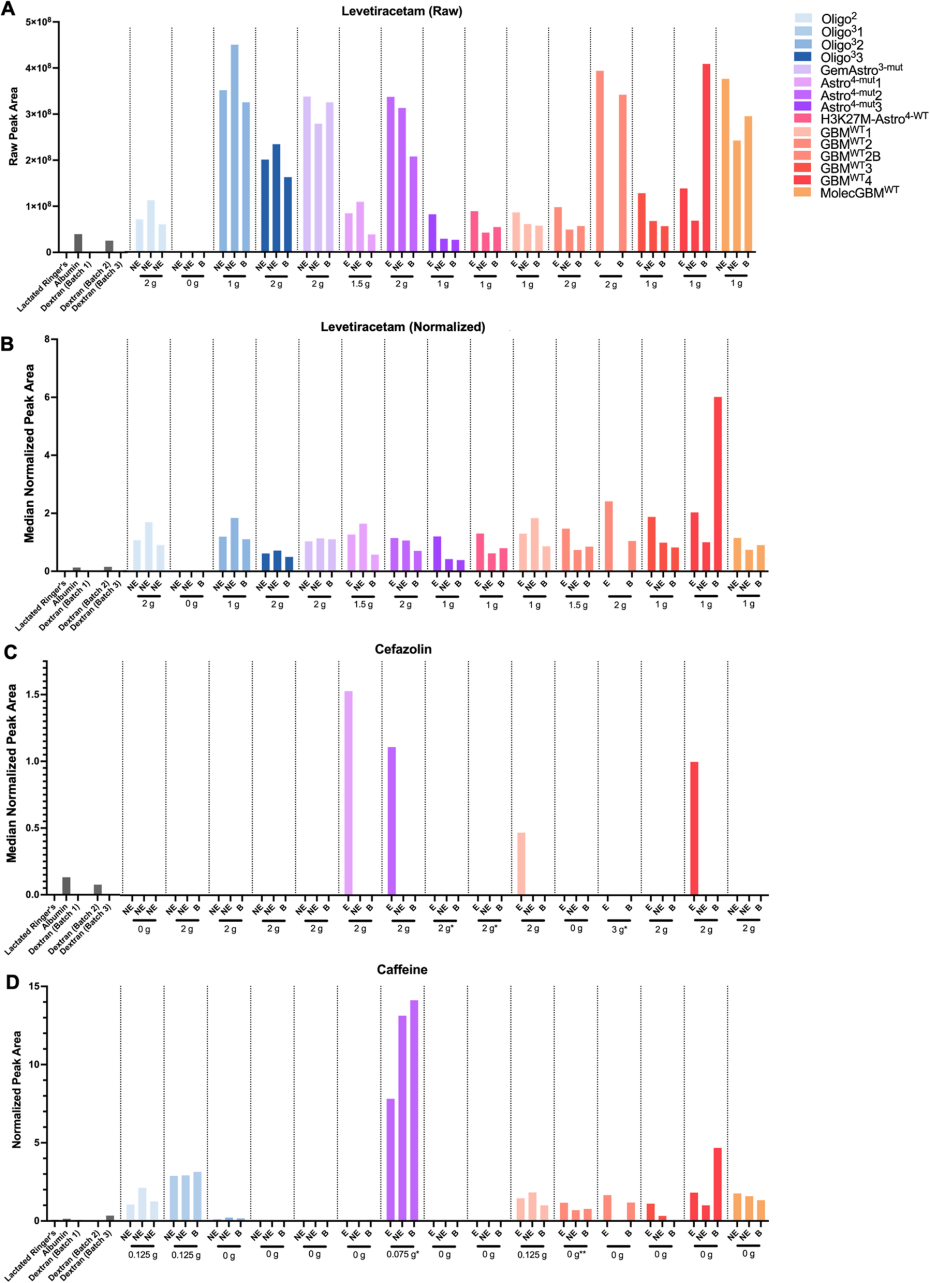
Peri-operatively administered levetiracetam, cefazolin, and caffeine were detected in microdialysates. Levetiracetam, an anti-epileptic drug (raw: **A**, normalized: **B**), cefazolin (**C**), and caffeine (**D**) were peri-operatively administered in certain patients and detected in one microdialysate aliquot. Recorded drug dosages are indicated for each patient; see Additional file 1: Table S1 for the timing between drug dosage and sampling via microdialysis for each patient. *administered, but not detected. **not administered, but detected

Levetiracetam (Keppra) is a CNS-penetrant anti-epileptic medication routinely administered to decrease the risk of intraoperative or postoperative seizures. Consistent with this, levetiracetam was detected in all catheters from 14/14 patients who received Keppra; no levetiracetam was detected in samples from patient Oligo^3^ did not receive it due to a reported allergy (see raw and batch-normalized data in Fig. 6A and B, respectively). Based on the raw data, levetiracetam levels varied by as much as 5.99 × levels within individual patients (Fig. 6A). Normalization visibly decreased the variance of Keppra levels within and between patients (Fig. 6B), consistent with the relatively arbitrary nature of numerical values obtained within any given batch. In contrast to levetiracetam, cefazolin (Ancef) is a preoperative antibiotic with poor CNS penetration. Accordingly, no detectable Ancef was present in microdialysate from in any catheter placed within brain or non-enhancing tumor tissue. However, Ancef was detected within microperfusate from the enhancing tumor catheter of four of the eight patients who received Ancef (Fig. 6C).

Mannitol is often administered intraoperatively to mitigate cerebral edema and brain swelling. Mannitol was only administered to 5 of 15 patients (Oligo^2^, Oligo^3^2, GemAstro^3−mut^, Astro^4−mut^1, and H3K27M-Astro^4−WT^). Mannitol peak areas were highest across all three catheters in these five patients when compared to those who were not administered mannitol (Additional file 1: Fig. S7A). However, minimal amounts of mannitol could be found in patients who did not receive it, suggesting baseline production within the CNS.

Additionally, six of the 15 surgeries were performed awake. Intravenous caffeine can be administered to aid in speech mapping. Caffeine levels were elevated in the four patients who received intravenous caffeine (Fig. 6D). Although patient GBM^WT^2 did not receive caffeine intraoperatively, raising the question of why we observed elevated caffeine levels in all three microdialysis catheters. However, when queried, the patient reported drinking a cup of black coffee in the early morning prior to admission for surgery. Detectable caffeine levels in four other patients for whom it was not administered prior to asleep surgery likely suggests consumption prior to admission for surgery. Additionally, acetaminophen is often administered during awake craniotomies to improve comfort. Intraoperatively administered acetaminophen could be quantified in the 6/6 patients who received it. It was detectable at lesser amounts in patient Astro^4−mut^1, potentially due to nondocumented preoperative administration (Additional file 1: Fig. S7B).

Collectively, these data suggest that acute intraoperative microdialysis paired with metabolomics may provide an efficient avenue to simultaneously evaluate relative extracellular free drug levels for multiple pharmacologic agents in multiple locations from only 20 μL of microdialysate.

## Discussion

We here report the caveats, mitigating strategies, and novel opportunities identified through our experience performing intra-operative microdialysis during human glioma resections. While salient findings of elevated tumor-associated guanidinoacetate and elevated to plasma-associated associated amino acids in the glioma extracellular microenvironment were previously reported [23], we here offer an uncensored view of our iterative learning process and the implications of lessons learned for future studies evaluating human glioma biology in situ.

### Optimization for the OR: high molecular weight catheters and elevated flow rates

Microdialysis is a technique that can be utilized to sample the extracellular interstitial fluid (ISF) of central nervous system (CNS) tissue, including tumors such as gliomas [7]. Prior microdialysis studies have utilized molecular weight cut-off catheters of 20 kDa [21, 31], which precludes sampling of larger analytes. Additionally, microdialysis is often performed post-operatively at a flow rate of 0.3–0.5 µL/min [13, 15, 17, 22]. While these slow flow rates and longer sampling time enable equilibration with the tissue interstitial fluid, this is not conducive to rapidly performing microdialysis in the intra-operative room when a patient’s CNS tissue is readily accessible. To understand gliomas in situ, we aimed to collect enough microdialysate from one patient to enable multi-omic analyses of their ISF. This required larger volumes and higher molecular weight cut-offs than would otherwise be afforded with slower flow rates. Moreover, we wished to maximize the range of soluble analytes captured. As such, obtaining an investigational device exemption for high molecular weight microdialysis catheters (< 100 kDa) and variable flow rate pumps enabled us to perform microdialysis during standard-of-care glioma resections.

### Patient selection and catheter placement

While HMW microdialysis was not technically challenging to perform during glioma resections, it did require thoughtful pre- and intra-operative planning. Tumor size and location determined whether intraoperative access could be available to multiple disparate tumor regions, including a region of relatively normal brain. We previously found that metabolic signatures tended to cluster by patient [23], meaning that an internal control of relatively uninvolved brain was required for each individual patient to meaningfully discern the tumor’s impact on the extracellular metabolome. Sufficient space was required within the surgical exposure for catheters sampling tumor and adjacent brain without impeding progress of the surgical resection. When microdialysis was performed during awake tumor resections, catheters were typically positioned away from eloquent regions so that stimulation mapping and dissection could progress unimpeded during the initial portions of the resection. Although the depth of catheter insertion was always planned to be a minimum of 10 mm to fully cover the membrane, suboptimal placement was noticed to render catheters liable to being bumped or dislodged, such that some portion of the membrane would be out of the tumor. Although the flexible tubing was tethered to improve stability, it was still relatively easy for a bumped catheter to inadvertently slip partially or completely out of position, requiring prompt reinsertion to avoid artifactually low metabolite recovery in the subsequent aliquot. For these pragmatic reasons, most patients enrolled in the intra-operative microdialysis protocol had relatively large tumors, frequently involving dominant frontal or temporal lobe requiring awake resection. Because catheters were quickly reinserted back into the tumor if they were dislodged, we have not found these reinsertions to have a significant impact on the microdialysate results.

Of note, patients with completely non-enhancing gliomas in this study tended to have relatively diffuse tumors, limiting availability of radiographically normal brain within the surgical exposure. In one case (Oligo^2^), the diffuse nature of the large tumor precluded sampling of uninvolved brain, preventing access to relatively un-involved brain, meaning that this patients’ samples could not be incorporated into analyses of the extracellular glioma metabolome. Conversely, tumor density played an unanticipated role in catheter placement that could not always be easily determined from the MRIs. Indeed, while the tumor of patient GemAstro^3−MUT^ had an enhancing component that we had planned on sampling, we were unable to insert the catheter into that portion of the tumor due to its high density and the flexible nature of the catheters. Instead, a second non-enhancing area was chosen in real-time using the probe registered to the neuronavigation system. Cases thereafter utilized a straight Rhoton microdissector registered to the neuronavigation system to create a path for the microdialysis catheters.

### From technical variables to functional insights

Several empiric decisions were required during the design of this study, including catheter size, type, as well as perfusate composition and flow rate. We intentionally incorporated flexibility into what was originally entitled a “feasibility” study, to enable iterative optimization based on accumulated experience. Indeed, based on the study’s intended exploratory nature, multiple parameters were changed throughout the trial. As such, the findings reported herein are not intended to serve as definitive statements on the impact of each parameter within intra-operative microdialysis studies. We simply aim to report on our experience and lessons learned with intra-operative microdialysis to date. Though LMW catheters were permitted, all cases used HMW catheters. Allowable lengths included 10, 20 and 30 mm. Preliminary findings did not demonstrate noticeably higher total metabolite recovery from 20 mm as compared to 10 mm catheters (Fig. 3), despite the softer and more flexible 20 mm catheters penetrating the tumor tissue less effectively. As such, we soon reverted to use of 10 mm catheters for all cases. Although flow rates from 0.3 to 2 µL/min were permitted, usable sample volumes and metabolite concentrations were obtained in initial patients at 2 µL/minute, and thus maintained for subsequent cases. It should be noted, however, that complete equilibration with the microenvironment may be more challenging at this higher flow rate, as illustrated by the still-declining 2-HG levels in sequential tumor aliquots for patient Astro^4−mut^1 (Additional file 1: Fig. S1) [24]. To help mitigate this impact in cross-patient comparisons, we utilized the 2nd aliquot after catheter implantation for all subsequent analyses.

The largest impact on results resulted from the perfusate fluid composition. Specifically, use of albumin rather than dextran as the oncotic agent increased abundance of several metabolites. Prior studies have utilized bovine serum albumin to decrease adherence of analytes to the microdialysis membrane [26]. However, clustering analyses via Pearson correlation revealed a marked impact of albumin on the recovered metabolome (Fig. 4A), with particularly elevated levels of medium and large fatty acids. Analysis of a flush sample demonstrated that these metabolites were present in the baseline sample, likely pre-bound to the utilized albumin, and did not reflect improved recovery from the tumor microenvironment via use of albumin-containing perfusate. While use a control flush sample should help mitigate perfusate-associated confounders, results obtained implicating metabolites present at baseline in albumin-containing perfusate should be regarded with caution. Moreover, the technical impact of albumin on metabolite quantification may warrant more detailed analysis.

Conversely, focal delivery of lactate via Lactated Ringer’s led to elevated recovery of both lactate and pyruvate. Drugs, such as cisplatin, have previously been delivered by reverse microdialysis into tumor and adjacent brain in patients with high-grade glioma—the extracellular metabolic impacts of which were evaluated in situ over multiple days [21]. We did not intend to perform reverse microdialysis with lactate, and had only chosen Lactated Ringer’s as the most isotonic solution readily available within the operating room. However, our findings suggest the feasibility of locally delivering a compound and evaluating its metabolic impacts within one surgery. We speculate that lower pyruvate levels in patient Oligo^2^ may suggest lower LDH activity in the only low-grade (WHO2) glioma among the 4 patients. Indeed, LDH has been reported as a as a negative prognostic indicator in higher-grade tumors [32, 33]. That this ability to modulate and functionally evaluate in situ metabolism was achievable in under 1 h during a standard of care glioma resection suggests exciting opportunities to interrogate other aspects of live human glioma metabolism in situ. Moreover, minimal changes in downstream derivatives of lactate and pyruvate, such as citrate in the TCA cycle, suggest that the timing at which metabolic changes are evaluated after focally delivery may be an important consideration in pharmacodynamic studies within patients. Concurrent systemic or focal delivery of stable isotope-labeled metabolites may directly demonstrate mechanisms of glioma metabolism and the differential impact of focally applied experimental perturbations.

### Batch effects

A limitation of untargeted metabolomic analysis is lack of absolute quantification. We previously utilized half of each aliquot from the first batch to perform targeted analysis of D- and L-2-HG—the sum of which demonstrated a linear correlation with the untargeted 2-HG raw peak area [23]. Although raw peak areas can enable relative quantification within batches, the same is not true between batches. However, normalization based on the sample’s median values within a batch may remove interesting sample-specific biology, as exemplified by the raw versus normalized peak areas for lactate. For this reason, the finding of abnormally elevated lactate or pyruvate in lactate-containing microdialysate samples could have been missed. This example illustrates the limitations of attempts to normalize samples across batch based on median values, if analytes are not equally represented across batches. To circumvent this problem in the future, all submissions moving forward will include one or more standard reference samples to facilitate cross-batch normalization to a common denominator.

### Multiplex drug detection

Within neuro-oncology, microdialysis has classically been utilized for pharmacokinetic studies of novel therapeutic agents in the post-operative setting [13, 21]. While multiple drugs are routinely administered during glioma resections, we had not specifically planned for drug analyses, given our use of untargeted metabolomics for discovery purposes and the relatively short intra-operative sampling time. We were pleasantly surprised to find that the metabolon annotations included multiple relevant pharmacologic agents. Levetiracetam was reliably detected in all samples of all patients who received it prior to surgery (41/41 samples), and none (0/3) samples from the one patient who did not receive the drug. We were surprised to find substantial variation in levetiracetam levels, as this drug is CNS penetrant and is presumed to equally penetrate brain and tumor. We speculate that observed variations may be attributable heterogeneous recovery across the catheters due to either variable diffusivity across heterogenous tumor regions or other technical factors. Additionally, although placement of intracerebral catheters has been reported to cause BBB disruption [34], we only identified the CNS-non-penetrant antibiotic, cefazolin in catheters placed within enhancing tissue, suggesting that the BBB disruption caused by catheter placement is less than that caused by the tumor. This finding is consistent with our previously reported identification of plasma-associated metabolites in microdialysate samples obtained from enhancing tumor regions [23]. Moreover, the lack of detection in the remaining four patients suggests that there may not have been sufficient BBB disruption to enable penetration of this antibiotic into the enhancing tumor, especially as cefazolin has a relatively short half-life of 4 h [35]. Moreover, untargeted metabolomics may not be optimized for cefazolin sensitivity.

As such, intra-operative microdialysis could provide a relevant tool to evaluate the relative CNS penetration of candidate therapeutic agents during phase 0 or window-of-opportunity studies following pre-operative drug administration. Importantly, while such studies are typically performed one drug at a time, our simultaneous detection of levetiracetam, Ancef, acetaminophen, mannitol, and caffeine suggest that neoadjuvant pharmacokinetic studies could be extended to drug cocktails rather than just individual drugs. While targeted analyses methods may require higher volume, concurrent use of more sensitive and quantitative targeted assays for known CNS-penetrant and non-penetrant drugs (such as levetiracetam and Ancef, respectively), may facilitate internal benchmarks to help evaluate the relative CNS penetration of novel agents. Future studies will further combine intra-operative microdialysis with serial plasma collection and post-microdialysis tissue sampling to help maximize translational insights from each surgery. Concurrent measurement of unbound plasma concentrations will be necessary as a comparison to the unbound ISF concentrations measured via microdialysis [36, 37].

### Recommendations for intra-operative microdialysis

Our intra-operative microdialysis trial has revealed numerous considerations for performing such studies: (1) the impact of perfusion fluid must be carefully considered prior to the case as downstream analyses may be impacted. We prefer to utilize 3% 500 kDa Dextran with artificial CSF across batches. (2) If a different perfusate is used, it is helpful to send a blank sample, prior to catheter insertion, to evaluate what background metabolites may be present within it. (3) Radiographical planning of the catheter locations ahead of time can maximize the efficiency of deploying this method within the operating room. However, alternative solutions based on live neuronavigation should be available in case catheter locations must change based on anatomy or tumor characteristics, including use of additional surgical tools to create a path into the tumor. (4) The use of reference sample sent across batches may be helpful for calibration across different runs, as relying on median normalization of the sample could artefactually minimize biology present within the tumor (e.g., lactate and pyruvate after focal delivery). (5) Extensive experimental and clinical documentation with case report forms and chart reviews greatly aids in maximizing consistency of methods across patients and interpretation of microdialysis data.

## Conclusion

In conclusion, intra-operative microdialysis is a powerful tool for querying and even dynamically interacting with the CNS extracellular microenvironment in situ, enabling global metabolomic analyses across diverse tissue regions with or without experimental modulation, while correlating to relative regional drug levels and extent of BBB disruption. We share our practical experience and lessons learned from fifteen cases to date, illustrating the impact of technical variables on results obtained.

While HMW microdialysis has been a powerful tool in the operating room for analysis of small molecules, including metabolites and certain drugs, recovery of more diverse analytes, including proteins and larger drugs, is precluded by the presence of a membrane. Moreover, this current study has demonstrated the potential analytical impacts of perfusate agents, including lactate and albumin. To that end, based on the lessons we have learned from our intraoperative microdialysis trial, are now developing a first-in-human microperfusion system that forgoes the semipermeable membrane and utilizes a dual push–pull pump system avoiding the need for potentially confounding oncotic agents such as albumin or dextran. We are hopeful such a system may empower recovery beyond metabolites and dialyzable drugs, to include lipids, proteins, extracellular vesicles, and cell-free DNA. Ultimately, we aim to deploy an expanding toolkit of methodologies to dynamically interact with the live human glioma with or without focal drug delivery, informing the iterative development and refinement of individualized therapeutic cocktails.

### Supplementary Information

**Additional file 1: Figure S1.** Time course of D-and L-2-hydroxyglutarate in sequential microdialysate aliquots from enhancing and non-enhancing tumor and brain. Microdialysate was collected in 20-min fractions at a flow rate of 2 uL/min from enhancing and non-enhancing tumor and brain adjacent to tumor in Patient Astro^4−mut^1, and subsequently quantified for D-and-L-2-hydroxyglutarate via mass spectrometry. **Figure S2**. High-molecular weight (100 kDa) microdialysis of CSF decreases the abundance of metabolites also found in CSF. CSF was microdialyzed with a 100 kDa microdialysis catheter, after which the CSF and microdialysate of CSF both underwent untargeted metabolomics via UPLC-MS/MS. Normalized peak areas for the 206 metabolites detected in both CSF and microdialysate of CSF are shown. **Figure S3.** Albumin perfusate elevates a small subset of metabolites in its microdialysates. The top 25 differentially abundant metabolites in the albumin-containing flush as compared to the dextran-containing flush in the second batch was used to generate a heatmap to evaluate the metabolic impacts of the albumin perfusate. **Figure S4**. Median normalization across batches normalizes lactate values across Lactated Ringer’s and non-Lactated Ringer’s-containing perfusates. Lactate was normalized across batches to have a median of n = 1; the median normalized peak areas for lactate are shown for all patients’ catheters and the flushes. **Figure S5.** Targeted versus untargeted quantification of lactate. Ten CSF samples underwent targeted lactate quantification to determine the relative performance of the untargeted metabolomics platform for relative lactate quantification, based on the linear correlation between targeted and untargeted metabolomics. **Figure S6.** Impact of lactate delivery on citrate in microdialysates. The raw peak area values for citrate, the derivative of pyruvate, were evaluated across microdialysates in which a Lactated Ringer’s-containing perfusate was or was not utilized. **Figure S7.** Detection of mannitol and acetaminophen via intra-operative microdialysis. Mannitol and acetaminophen were detected via untargeted metabolomics (UPLC-MS/MS) of intra-operatively acquired microdialysates. Known drug dosages were collected from patients’ charts; timing between catheter sampling and dose administration are found in Table S1. **Table S1.** Timing of drug administration relative to catheter sampling for drugs detected via microdialysis.

## Data Availability

All data are available as supplementary files in the prior publication,
