# Supplementary material for: Methodological and analytical considerations for intra-operative microdialysis
Source: Fluids Barriers CNS. 2023 Dec 19;20:94. doi: 10.1186/s12987-023-00497-2 (PMC10729367; doi:10.1186/s12987-023-00497-2)
Supplement: Supplementary file 1 — Additional file 1: Figure S1. Time course of D-and L-2-hydroxyglutarate in sequential microdialysate aliquots from enhancing and non-enhancing tumor and brain. Microdialysate was collected in 20-min fractions at a flow rate of 2 uL/min from enhancing and non-enhancing tumor and brain adjacent to tumor in Patient Astro4−mut1, and subsequently quantified for D-and-L-2-hydroxyglutarate via mass spectrometry. Figure S2. High-molecular weight (100 kDa) microdialysis of CSF decreases the abundance of metabolites also found in CSF. CSF was microdialyzed with a 100 kDa microdialysis catheter, after which the CSF and microdialysate of CSF both underwent untargeted metabolomics via UPLC-MS/MS. Normalized peak areas for the 206 metabolites detected in both CSF and microdialysate of CSF are shown. Figure S3. Albumin perfusate elevates a small subset of metabolites in its microdialysates. The top 25 differentially abundant metabolites in the albumin-containing flush as compared to the dextran-containing flush in the second batch was used to generate a heatmap to evaluate the metabolic impacts of the albumin perfusate. Figure S4. Median normalization across batches normalizes lactate values across Lactated Ringer’s and non-Lactated Ringer’s-containing perfusates. Lactate was normalized across batches to have a median of n = 1; the median normalized peak areas for lactate are shown for all patients’ catheters and the flushes. Figure S5. Targeted versus untargeted quantification of lactate. Ten CSF samples underwent targeted lactate quantification to determine the relative performance of the untargeted metabolomics platform for relative lactate quantification, based on the linear correlation between targeted and untargeted metabolomics. Figure S6. Impact of lactate delivery on citrate in microdialysates. The raw peak area values for citrate, the derivative of pyruvate, were evaluated across microdialysates in which a Lactated Ringer’s-containing perfusate was or was not utilized. F [file 12987_2023_497_MOESM1_ESM.docx]

**Additional file**

**Additional Figures**


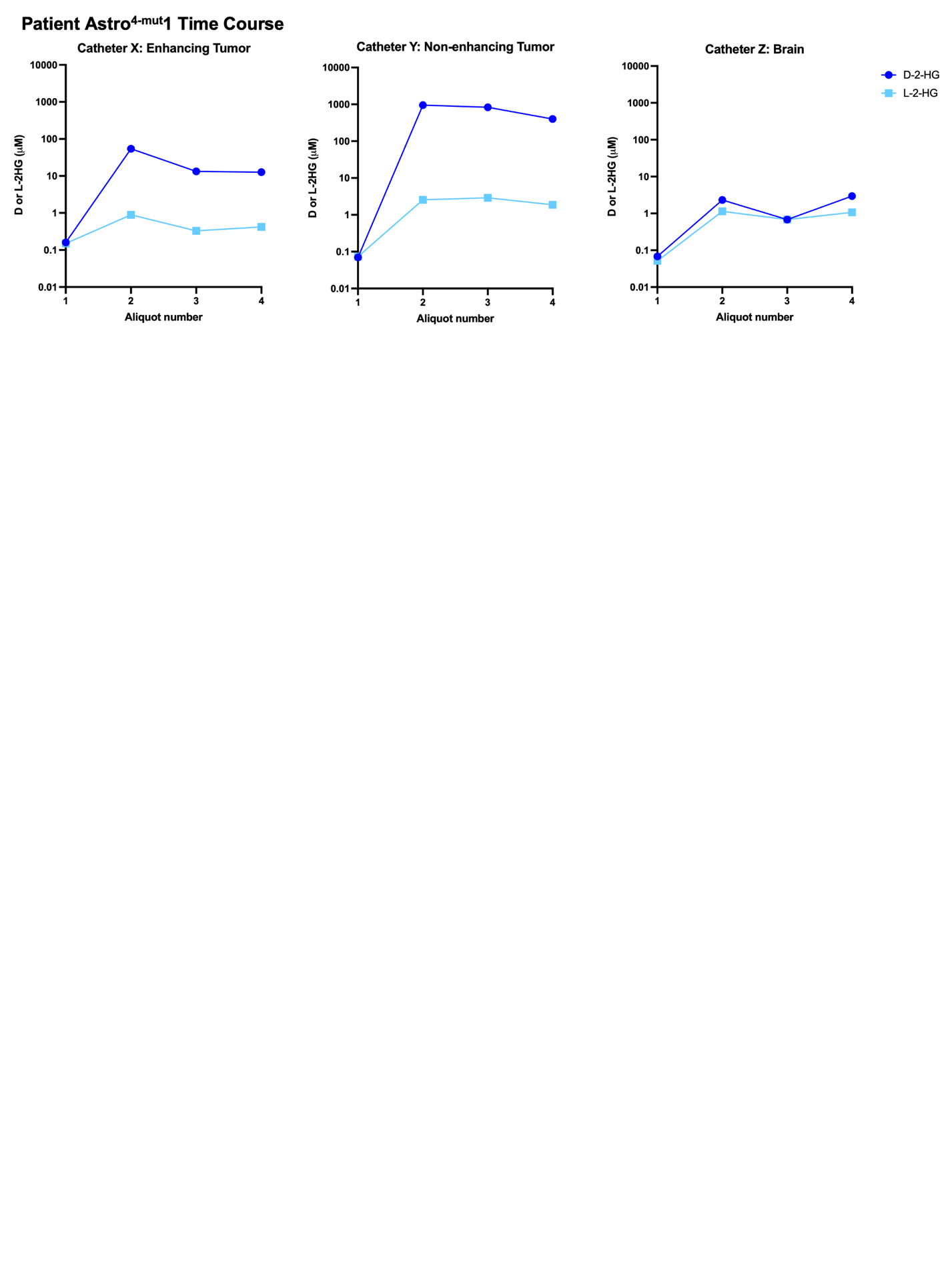
**Figure S1. Time course of D-and L-2-hydroxyglutarate in sequential microdialysate aliquots from enhancing and non-enhancing tumor and brain.** Microdialysate was collected in 20-minute fractions at a flow rate of 2 uL/min from enhancing and non-enhancing tumor and brain adjacent to tumor in Patient Astro^4-mut^1, and subsequently quantified for D-and-L-2-hydroxyglutarate via mass spectrometry.


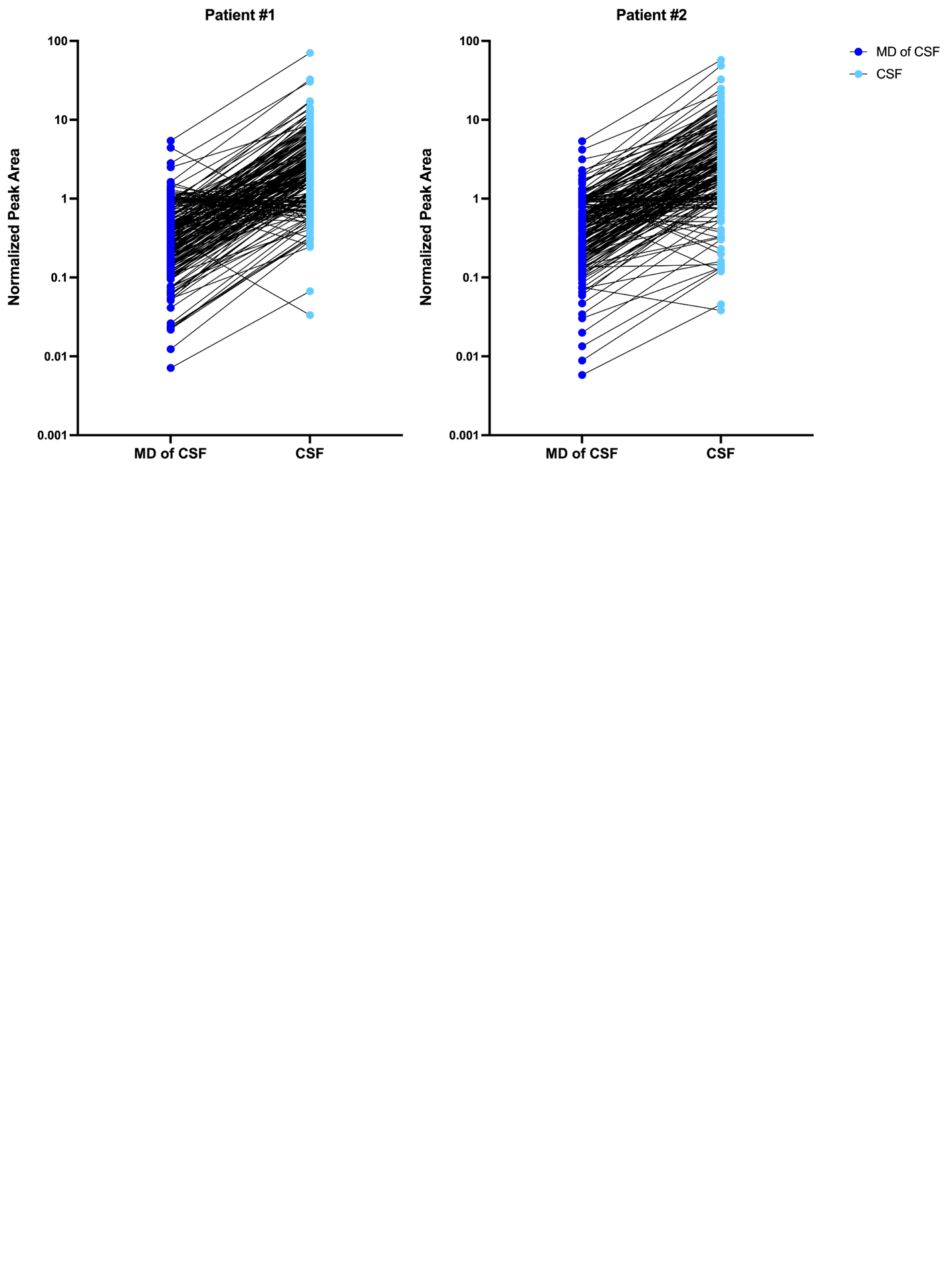
**Figure S2**. **High-molecular weight (100 kDa) microdialysis of CSF decreases the abundance of metabolites also found in CSF.**

CSF was microdialyzed with a 100 kDa microdialysis catheter, after which the CSF and microdialysate of CSF both underwent untargeted metabolomics via UPLC-MS/MS. Normalized peak areas for the 206 metabolites detected in both CSF and microdialysate of CSF are shown.


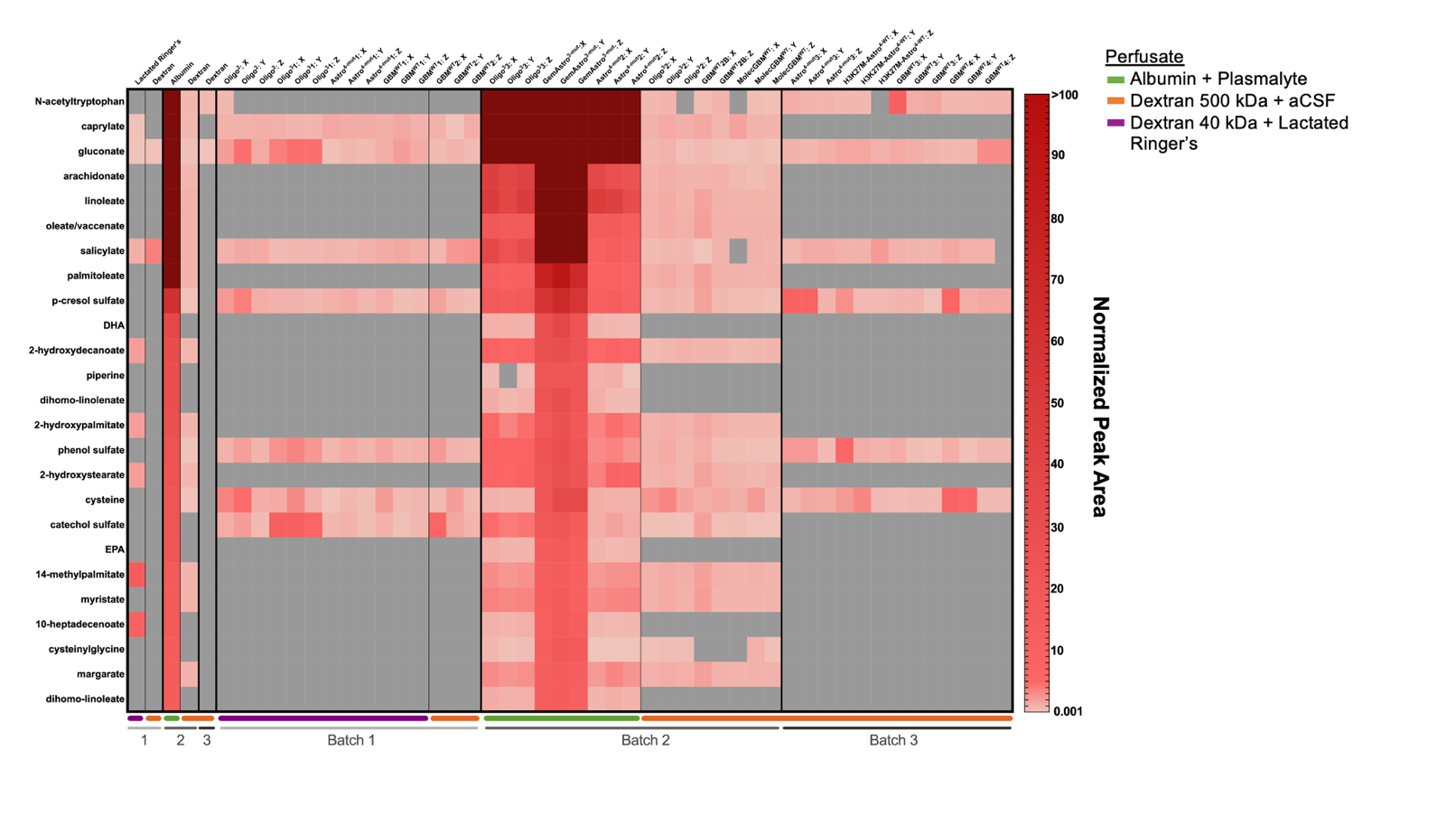
**Figure S3. Albumin perfusate elevates a small subset of metabolites in its microdialysates.**

The top 25 differentially abundant metabolites in the albumin-containing flush as compared to the dextran-containing flush in the second batch was used to generate a heatmap to evaluate the metabolic impacts of the albumin perfusate.


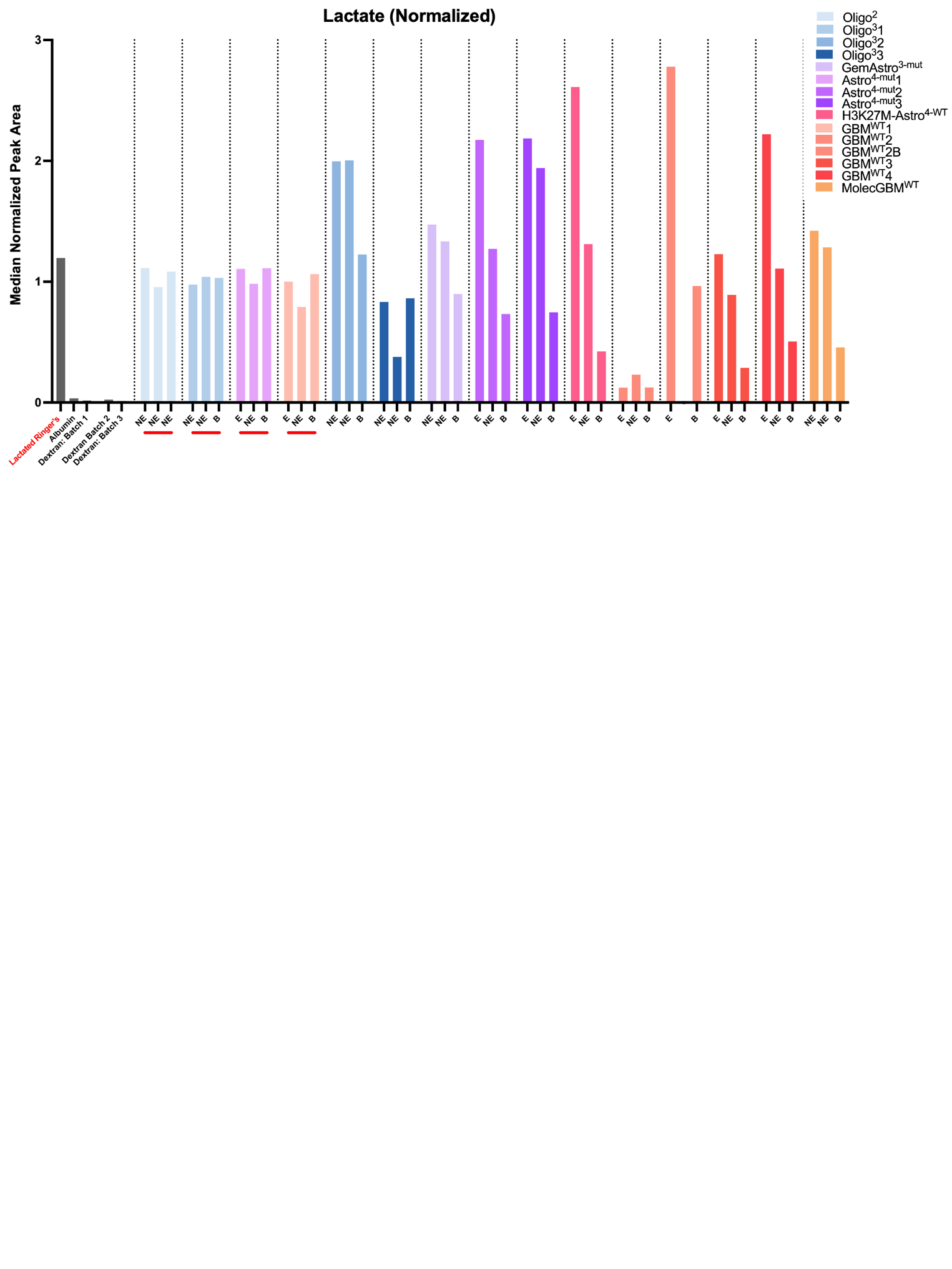
**Figure S4**. **Median normalization across batches normalizes lactate values across Lactated Ringer’s and non-Lactated Ringer’s-containing perfusates.**

Lactate was normalized across batches to have a median of n=1; the median normalized peak areas for lactate are shown for all patients’ catheters and the flushes.

**Figure S5. Targeted versus untargeted quantification of lactate.**


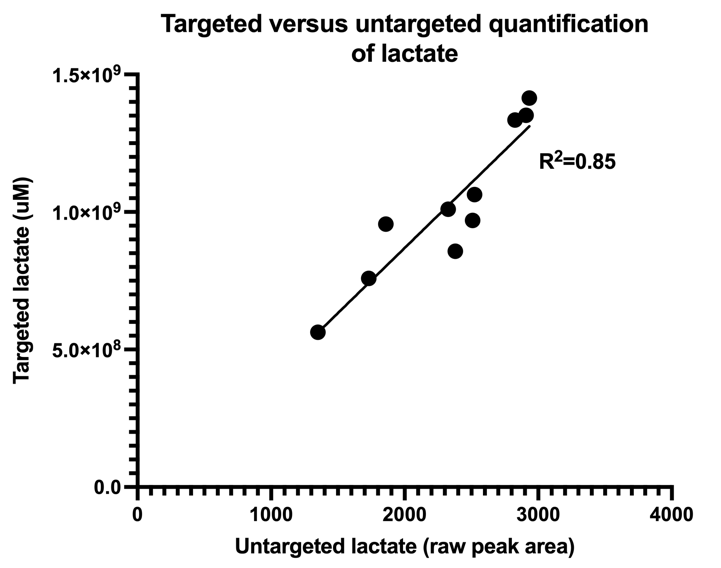


Ten CSF samples underwent targeted lactate quantification to determine the relative performance of the untargeted metabolomics platform for relative lactate quantification, based on the linear correlation between targeted and untargeted metabolomics.

**Figure S6. Impact of lactate delivery on citrate in microdialysates.**

**
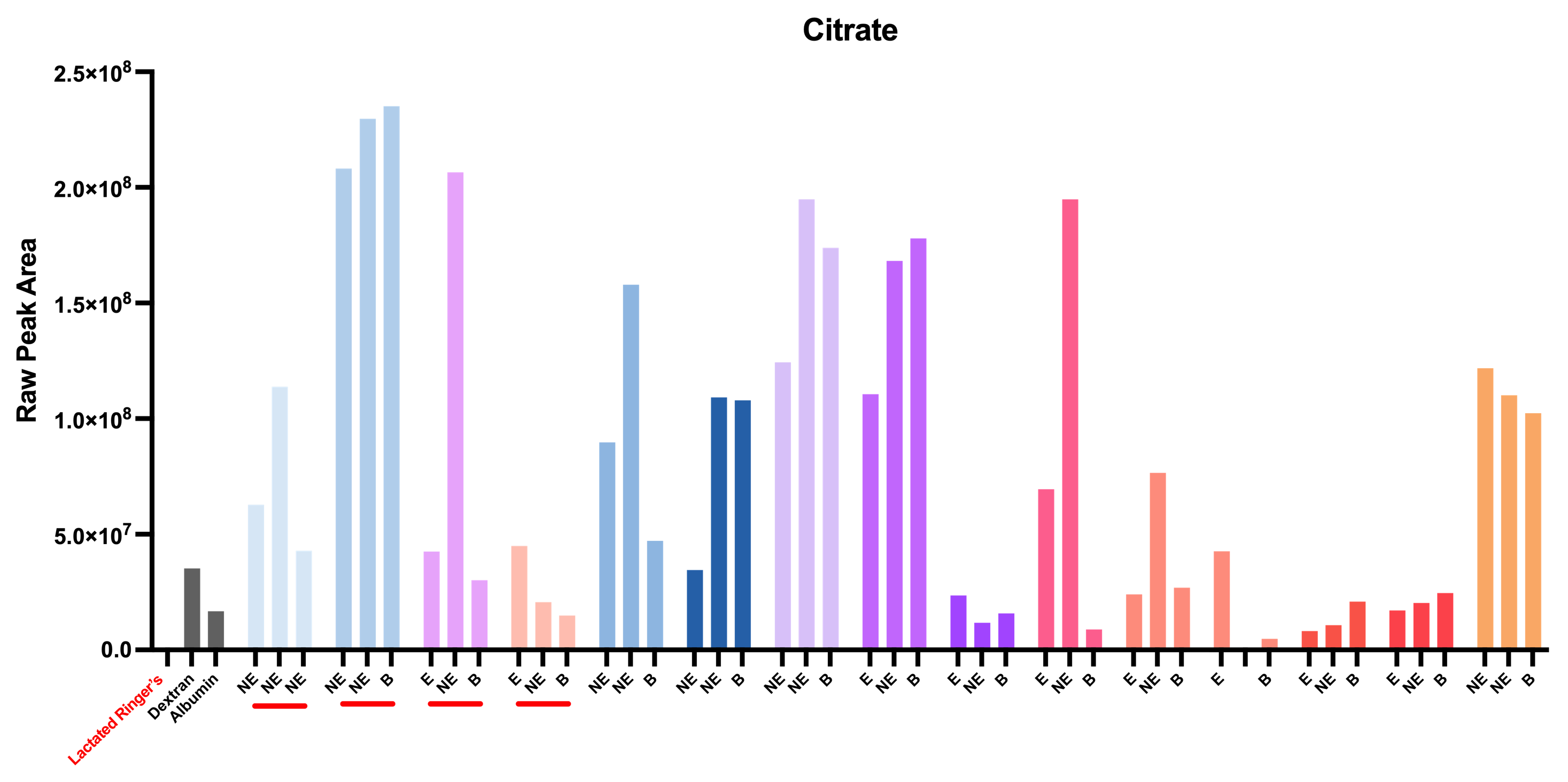
**

The raw peak area values for citrate, the derivative of pyruvate, were evaluated across microdialysates in which a Lactated Ringer’s-containing perfusate was or was not utilized.

**Figure S7. Detection of mannitol and acetaminophen via intra-operative microdialysis.**


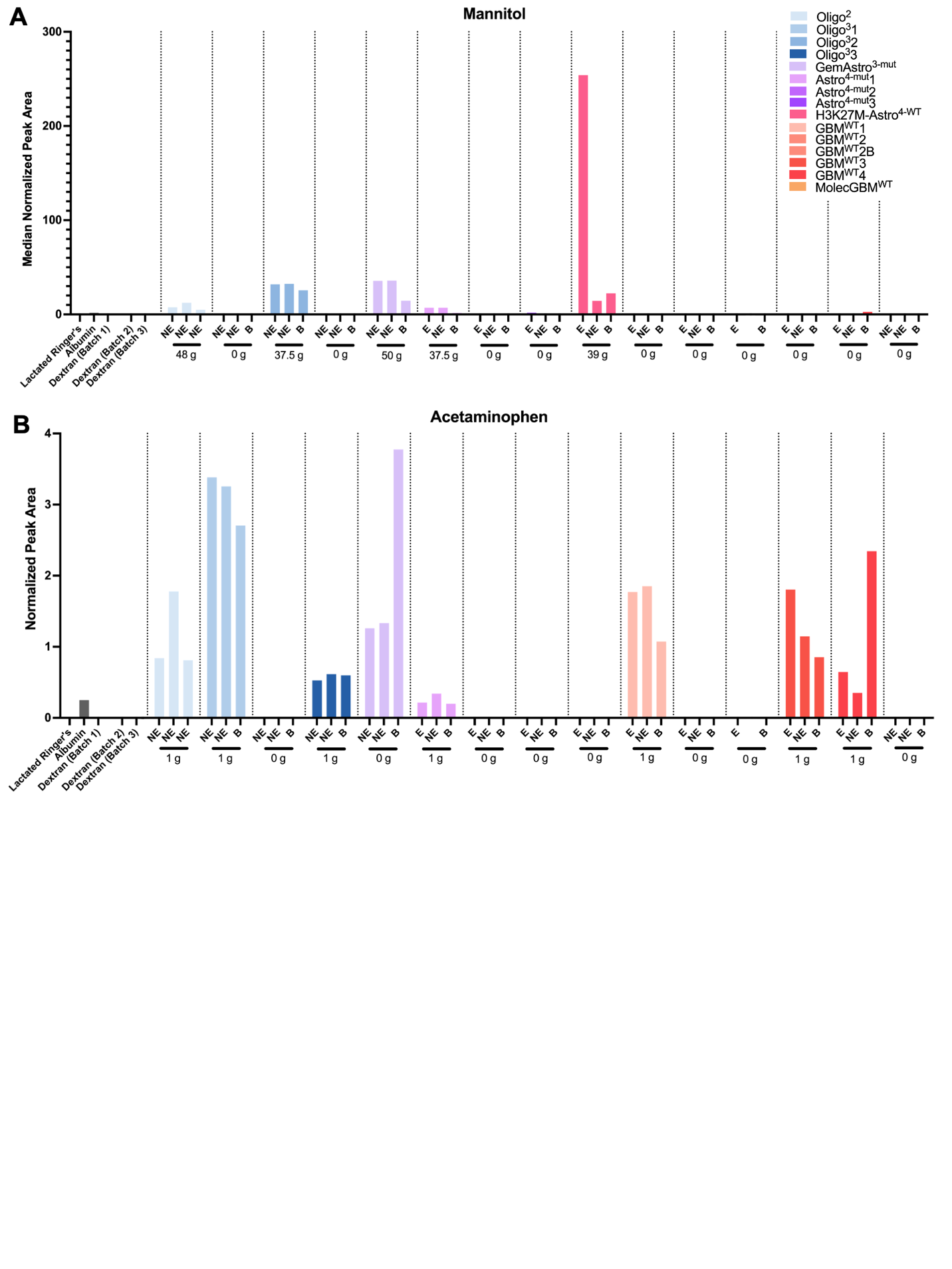
Mannitol and acetaminophen were detected via untargeted metabolomics (UPLC-MS/MS) of intra-operatively acquired microdialysates. Known drug dosages were collected from patients’ charts; timing between catheter sampling and dose administration are found in Table S1.

**Additional Tables**

**Table S1.** Timing of drug administration relative to catheter sampling for drugs detected via microdialysis.

|  | Time between catheter sampling and dose administration | | | | |
| --- | --- | --- | --- | --- | --- |
| Patient ID | Ancef | Levetiracetam | Mannitol | Acetaminophen | Caffeine |
| Oligo^2^ | N/A | 1.5 g ~2 hours;  0.5 g bolus during | 50 min | ~10 min | During |
| Oligo^3^1 | During | N/A | N/A | ~1.5 hours | ~1 hour |
| Oligo^3^2 | ~2.5 hours | ~3.2 hours | ~2.3 hours | N/A | N/A |
| Oligo^3^3 | ~10 min | Unknown | N/A | ~5 hours | N/A |
| GemAstro^3-mut^ | ~2 hours | ~2.8 hours | ~2.5 hours | N/A | N/A |
| Astro^4-mut^1 | ~1 hour | ~4.2 hours | ~4 hours | ~7.3 hours | N/A |
| Astro^4-mut^2 | ~20 min | ~3.2 hours | N/A | N/A | During |
| Astro^4-mut^3 | ~2.5 hours | ~2.7 hours | N/A | N/A | N/A |
| H3K27M-Astro^4-WT^ | ~1.8 hours | ~2.8 hours | ~1.5 hours | N/A | N/A |
| GBM^WT^1 | ~2.2 hours | ~2.8 hours | N/A | ~1 hour | ~10 min |
| GBM^WT^2 | N/A | 1.5 g ~3.2 hours  0.5 g bolus during | N/A | N/A | N/A |
| GBM^WT^2B | ~2.2 hours | ~2.2 hours | N/A | N/A | N/A |
| GBM^WT^3 | ~20 min | ~3.2 hours | N/A | ~5.2 hours | N/A |
| GBM^WT^4 | ~30 min | ~3.3 hours | N/A | ~7.3 hours | N/A |
| MolecGBM^WT^ | ~2 hours | ~2.3 hours | N/A | N/A | N/A |
